# Di-μ-methanolato-κ^4^ *O*:*O*-bis[tri­chlorido(dimethyl­formamide-κ*O*)tin(IV)]

**DOI:** 10.1107/S1600536810053997

**Published:** 2011-01-08

**Authors:** Qijun Zhang, Handong Yin, Daqi Wang

**Affiliations:** a College of Chemistry and Chemical Engineering, Liaocheng University, Shandong 252059, People’s Republic of China

## Abstract

The title compound, [Sn_2_(CH_3_O)_2_Cl_6_(C_3_H_7_NO)_2_], contains two hexa­coordinated Sn^IV^ atoms symmetrically bridged by two deprotonated methanol ligands, with an inversion center in the middle of the planar Sn_2_O_2_ ring. The other sites of the distorted octa­hedral coordination geometry of the Sn^IV^ atom are occupied by three Cl atoms and one O atom from a dimethyl­formamide mol­ecule. The complex mol­ecules are connected by weak C—H⋯Cl hydrogen bonds into a two-dimensional supra­molecular network parallel to (10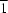).

## Related literature

For related tin(IV) compounds, see: Mao & You (1990 ▶); Reuter & Schröder (1992 ▶).
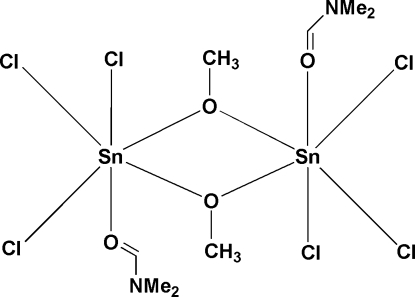


## Experimental

### 

#### Crystal data

[Sn_2_(CH_3_O)_2_Cl_6_(C_3_H_7_NO)_2_]
*M* *_r_* = 658.34
Monoclinic, 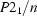

*a* = 8.589 (8) Å
*b* = 11.4444 (13) Å
*c* = 11.8453 (10) Å
β = 111.155 (1)°
*V* = 1085.9 (10) Å^3^
*Z* = 2
Mo *K*α radiation
μ = 3.05 mm^−1^
*T* = 298 K
0.22 × 0.17 × 0.16 mm

#### Data collection

Bruker APEX CCD diffractometer
Absorption correction: multi-scan (*SADABS*; Sheldrick, 1996 ▶) *T*_min_ = 0.553, *T*_max_ = 0.641
5512 measured reflections
1903 independent reflections
1554 reflections with *I* > 2σ(*I*)
*R*_int_ = 0.063

#### Refinement

*R*[*F* ^2^ > 2σ(*F* ^2^)] = 0.044
*wR*(*F* ^2^) = 0.117
*S* = 1.14
1903 reflections
103 parameters
13 restraints
H-atom parameters constrained
Δρ_max_ = 1.54 e Å^−3^
Δρ_min_ = −2.07 e Å^−3^

### 

Data collection: *SMART* (Bruker, 2007 ▶); cell refinement: *SAINT* (Bruker, 2007 ▶); data reduction: *SAINT*; program(s) used to solve structure: *SHELXS97* (Sheldrick, 2008 ▶); program(s) used to refine structure: *SHELXL97* (Sheldrick, 2008 ▶); molecular graphics: *SHELXTL* (Sheldrick, 2008 ▶) and *X-SEED* (Barbour, 2001 ▶); software used to prepare material for publication: *SHELXTL*.

## Supplementary Material

Crystal structure: contains datablocks I, global. DOI: 10.1107/S1600536810053997/hy2391sup1.cif

Structure factors: contains datablocks I. DOI: 10.1107/S1600536810053997/hy2391Isup2.hkl

Additional supplementary materials:  crystallographic information; 3D view; checkCIF report

## Figures and Tables

**Table 1:** Selected bond lengths (Å)

Sn1—O1	2.106 (5)
Sn1—O1^i^	2.101 (5)
Sn1—O2	2.108 (4)
Sn1—Cl1	2.372 (2)
Sn1—Cl2	2.3743 (18)
Sn1—Cl3	2.368 (2)

Symmetry code: (i) 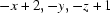.

**Table 2:** Hydrogen-bond geometry (Å, °)

*D*—H⋯*A*	*D*—H	H⋯*A*	*D*⋯*A*	*D*—H⋯*A*
C1—H1*C*⋯Cl3	0.96	2.72	3.356 (8)	124
C3—H3*A*⋯Cl3^ii^	0.96	2.95	3.895 (11)	170
C4—H4*B*⋯Cl1^iii^	0.96	2.90	3.837 (9)	164

Symmetry codes: (ii) 
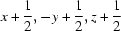
; (iii) 
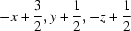
.

**Fig. 1.**
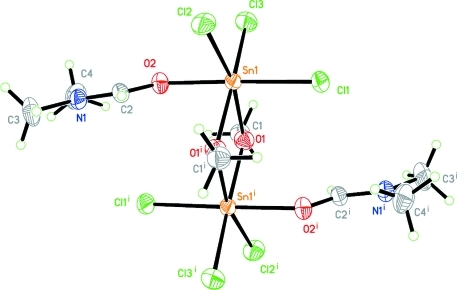
The molecular structure of the title compound, with 50% probability displacement ellipsoids. [Symmetry code: (i) -x+2, -y, -z+1.]

**Fig. 2.**
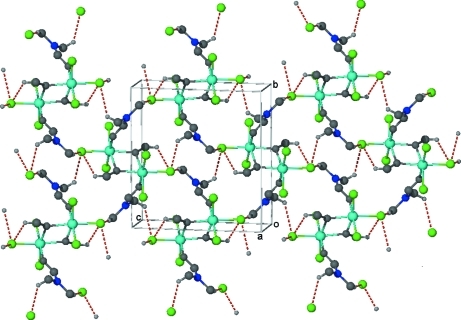
A view of the two-dimensional polymer-like supramolecular network in the title compound.

## supplementary crystallographic information

### Comment

The title compound (Fig.1) consists of six Cl anions, two methoxo anions, two Sn^IV^ ions and two dimethylformamide molecules. The molecule has an inversion center in the middle of the Sn_2_O_2_ ring. This ring is planar and can be described as rhombic, with the endocyclic angles at the O atoms larger than those at the Sn atoms [Sn1^i^—O1—Sn1 = 106.9 (2), O1^i^—Sn1—O1 = 73.1 (2)°. Symmetry code: (i) -x+2, -y, -z+1]. The Sn1—O1 distance [2.106 (5) Å] is very close to the Sn1—O1^i^ distance [2.101 (5) Å] (Table 1). Each Sn^IV^ atom is hexacoordinated with two methoxo anions, three Cl anions and one dimethylformamide molecule in a distorted octahedral geometry.

As is indicated from Fig. 2 and Table 2, the intramolecular interactions, C1—H1C···Cl3, strengthen the dimeric unit and the intermolecular ones, C3—H3A···Cl3^ii^ and C4—H4B···Cl1^iii^ [symmetry codes: (ii) x+1/2, -y+1/2, z+1/2; (iii) -x+3/2, y+1/2, -z+1/2], give rise to a two-dimensional polymer-like supramolecular network.

### Experimental

Stannic chloride hydrate (0.4 mmol, 0.14 g) was dissolved in methanol (20 ml) and dimethylformamide (5 ml) was added with stirring at room temperature. The mixture was allowed to react for 6 h and was then filtered. Colorless crystals suitable for X-ray analysis were obtained by slow evaporation of methanol over a period of two weeks (yield: 60%). Analysis, calculated for C_8_H_20_Cl_6_N_2_O_4_Sn_2_: C 14.59, H 3.06, N 4.25%; found: C 14.58, H 3.04, N 4.27%.

### Refinement

H atoms were placed in calculated positions and treated as riding on their parent atoms, with C—H = 0.93 (CH) and 0.96 (CH_3_) Å and with *U*_iso_(H) = 1.2(1.5 for methyl)*U*_eq_(C). The highest residual electron density was found at 0.72 Å from H1A atom and the deepest hole at 1.01 Å from Sn1 atom.

### Crystal data

**Table:**

[Sn_2_(CH_3_O)_2_Cl_6_(C_3_H_7_NO)_2_]	*F*(000) = 632
*M**_r_* = 658.34	*D*_x_ = 2.013 Mg m^−^^3^
Monoclinic, *P*2_1_/*n*	Mo *K*α radiation, λ = 0.71073 Å
Hall symbol: -P 2yn	Cell parameters from 2694 reflections
*a* = 8.589 (8) Å	θ = 2.6–27.0°
*b* = 11.4444 (13) Å	µ = 3.05 mm^−^^1^
*c* = 11.8453 (10) Å	*T* = 298 K
β = 111.155 (1)°	Block, colourless
*V* = 1085.9 (10) Å^3^	0.22 × 0.17 × 0.16 mm
*Z* = 2	

### Data collection

**Table:**

Bruker APEX CCD diffractometer	1903 independent reflections
Radiation source: fine-focus sealed tube	1554 reflections with *I* > 2σ(*I*)
graphite	*R*_int_ = 0.063
φ and ω scans	θ_max_ = 25.0°, θ_min_ = 2.6°
Absorption correction: multi-scan (*SADABS*; Sheldrick, 1996)	*h* = −6→10
*T*_min_ = 0.553, *T*_max_ = 0.641	*k* = −13→12
5512 measured reflections	*l* = −14→14

### Refinement

**Table:**

Refinement on *F*^2^	Primary atom site location: structure-invariant direct methods
Least-squares matrix: full	Secondary atom site location: difference Fourier map
*R*[*F*^2^ > 2σ(*F*^2^)] = 0.044	Hydrogen site location: inferred from neighbouring sites
*wR*(*F*^2^) = 0.117	H-atom parameters constrained
*S* = 1.14	*w* = 1/[σ^2^(*F*_o_^2^) + (0.065*P*)^2^] where *P* = (*F*_o_^2^ + 2*F*_c_^2^)/3
1903 reflections	(Δ/σ)_max_ = 0.012
103 parameters	Δρ_max_ = 1.54 e Å^−^^3^
13 restraints	Δρ_min_ = −2.07 e Å^−^^3^

### Fractional atomic coordinates and isotropic or equivalent isotropic displacement parameters (Å^2^)

**Table:**

	*x*	*y*	*z*	*U*_iso_*/*U*_eq_	
Sn1	0.99552 (5)	0.07346 (3)	0.37511 (4)	0.0383 (2)	
Cl1	1.1769 (3)	−0.06088 (15)	0.3320 (2)	0.0619 (5)	
Cl2	1.1500 (2)	0.24014 (15)	0.35788 (19)	0.0586 (5)	
Cl3	0.7989 (3)	0.07190 (15)	0.17440 (19)	0.0629 (5)	
N1	0.7652 (7)	0.3252 (5)	0.5241 (5)	0.0498 (14)	
O1	0.8762 (6)	−0.0591 (4)	0.4372 (5)	0.0511 (10)	
O2	0.8269 (5)	0.1862 (4)	0.4134 (4)	0.0461 (10)	
C1	0.7018 (10)	−0.0904 (6)	0.3757 (7)	0.0556 (11)	
H1A	0.6323	−0.0359	0.3974	0.083*	
H1B	0.6828	−0.1677	0.3993	0.083*	
H1C	0.6750	−0.0881	0.2897	0.083*	
C2	0.8701 (8)	0.2657 (5)	0.4921 (6)	0.0438 (15)	
H2	0.9832	0.2824	0.5290	0.053*	
C3	0.8233 (14)	0.4147 (7)	0.6175 (10)	0.078 (3)	
H3A	0.9420	0.4236	0.6411	0.117*	
H3B	0.7968	0.3919	0.6864	0.117*	
H3C	0.7696	0.4876	0.5864	0.117*	
C4	0.5869 (9)	0.3028 (7)	0.4732 (9)	0.072 (2)	
H4A	0.5679	0.2218	0.4517	0.109*	
H4B	0.5372	0.3501	0.4023	0.109*	
H4C	0.5377	0.3217	0.5319	0.109*	

### Atomic displacement parameters (Å^2^)

**Table:**

	*U*^11^	*U*^22^	*U*^33^	*U*^12^	*U*^13^	*U*^23^
Sn1	0.0438 (3)	0.0350 (3)	0.0425 (3)	0.00087 (17)	0.0231 (2)	0.00141 (16)
Cl1	0.0809 (14)	0.0551 (10)	0.0707 (13)	0.0188 (9)	0.0527 (11)	0.0088 (8)
Cl2	0.0610 (11)	0.0488 (9)	0.0764 (13)	−0.0084 (8)	0.0371 (10)	0.0094 (8)
Cl3	0.0709 (13)	0.0691 (12)	0.0452 (10)	0.0061 (9)	0.0165 (9)	0.0006 (8)
N1	0.056 (4)	0.044 (3)	0.059 (4)	0.008 (3)	0.032 (3)	0.000 (3)
O1	0.0531 (15)	0.0515 (15)	0.0514 (15)	−0.0033 (12)	0.0221 (12)	0.0004 (12)
O2	0.043 (2)	0.044 (2)	0.056 (3)	−0.001 (2)	0.023 (2)	−0.008 (2)
C1	0.0560 (14)	0.0550 (13)	0.0553 (14)	−0.0025 (9)	0.0195 (9)	0.0009 (9)
C2	0.048 (4)	0.044 (3)	0.044 (3)	0.003 (3)	0.022 (3)	−0.004 (3)
C3	0.097 (7)	0.065 (5)	0.083 (7)	0.001 (4)	0.046 (6)	−0.030 (4)
C4	0.051 (5)	0.071 (5)	0.099 (7)	0.016 (4)	0.031 (4)	0.000 (5)

### Geometric parameters (Å, °)

**Table:**

Sn1—O1	2.106 (5)	O2—C2	1.259 (7)
Sn1—O1^i^	2.101 (5)	C1—H1A	0.9600
Sn1—O2	2.108 (4)	C1—H1B	0.9600
Sn1—Cl1	2.372 (2)	C1—H1C	0.9600
Sn1—Cl2	2.3743 (18)	C2—H2	0.9300
Sn1—Cl3	2.368 (2)	C3—H3A	0.9600
N1—C2	1.291 (8)	C3—H3B	0.9600
N1—C4	1.452 (9)	C3—H3C	0.9600
N1—C3	1.457 (10)	C4—H4A	0.9600
O1—C1	1.455 (9)	C4—H4B	0.9600
O1—Sn1^i^	2.101 (5)	C4—H4C	0.9600
			
O1^i^—Sn1—O2	87.65 (18)	O1—C1—H1A	109.5
O1^i^—Sn1—O1	73.1 (2)	O1—C1—H1B	109.5
O2—Sn1—O1	84.69 (19)	H1A—C1—H1B	109.5
O1^i^—Sn1—Cl3	166.73 (15)	O1—C1—H1C	109.5
O2—Sn1—Cl3	85.64 (14)	H1A—C1—H1C	109.5
O1—Sn1—Cl3	94.86 (16)	H1B—C1—H1C	109.5
O1^i^—Sn1—Cl1	92.45 (15)	O2—C2—N1	123.2 (6)
O2—Sn1—Cl1	177.31 (12)	O2—C2—H2	118.4
O1—Sn1—Cl1	92.76 (15)	N1—C2—H2	118.4
Cl3—Sn1—Cl1	93.72 (9)	N1—C3—H3A	109.5
O1^i^—Sn1—Cl2	93.32 (14)	N1—C3—H3B	109.5
O2—Sn1—Cl2	88.59 (13)	H3A—C3—H3B	109.5
O1—Sn1—Cl2	165.05 (15)	N1—C3—H3C	109.5
Cl3—Sn1—Cl2	97.94 (8)	H3A—C3—H3C	109.5
Cl1—Sn1—Cl2	94.09 (8)	H3B—C3—H3C	109.5
C2—N1—C4	121.9 (6)	N1—C4—H4A	109.5
C2—N1—C3	120.6 (7)	N1—C4—H4B	109.5
C4—N1—C3	117.4 (6)	H4A—C4—H4B	109.5
C1—O1—Sn1^i^	124.2 (4)	N1—C4—H4C	109.5
C1—O1—Sn1	123.0 (4)	H4A—C4—H4C	109.5
Sn1^i^—O1—Sn1	106.9 (2)	H4B—C4—H4C	109.5
C2—O2—Sn1	124.0 (4)		
			
O1^i^—Sn1—O1—C1	154.0 (6)	Cl2—Sn1—O1—Sn1^i^	−25.5 (7)
O2—Sn1—O1—C1	64.8 (5)	O1^i^—Sn1—O2—C2	42.9 (5)
Cl3—Sn1—O1—C1	−20.3 (5)	O1—Sn1—O2—C2	116.2 (5)
Cl1—Sn1—O1—C1	−114.3 (5)	Cl3—Sn1—O2—C2	−148.5 (5)
Cl2—Sn1—O1—C1	128.5 (6)	Cl2—Sn1—O2—C2	−50.4 (5)
O1^i^—Sn1—O1—Sn1^i^	0.0	Sn1—O2—C2—N1	−171.9 (5)
O2—Sn1—O1—Sn1^i^	−89.1 (2)	C4—N1—C2—O2	1.6 (10)
Cl3—Sn1—O1—Sn1^i^	−174.30 (17)	C3—N1—C2—O2	178.8 (7)
Cl1—Sn1—O1—Sn1^i^	91.72 (19)		

Symmetry codes: (i) −*x*+2, −*y*, −*z*+1.

### Hydrogen-bond geometry (Å, °)

**Table:**

*D*—H···*A*	*D*—H	H···*A*	*D*···*A*	*D*—H···*A*
C1—H1C···Cl3	0.96	2.72	3.356 (8)	124
C3—H3A···Cl3^ii^	0.96	2.95	3.895 (11)	170
C4—H4B···Cl1^iii^	0.96	2.90	3.837 (9)	164

Symmetry codes: (ii) *x*+1/2, −*y*+1/2, *z*+1/2; (iii) −*x*+3/2, *y*+1/2, −*z*+1/2.
